# Supplementary material for: NS5A domain I antagonises PKR to facilitate the assembly of infectious hepatitis C virus particles
Source: PLoS Pathog. 2023 Feb 16;19(2):e1010812. doi: 10.1371/journal.ppat.1010812 (PMC9977016; doi:10.1371/journal.ppat.1010812)
Supplement: S1 Fig — In vitro transcribed mSGR-luc-JFH-1 RNAs containing the indicated mutations were electroporated into (A) Huh7 and (B) Huh7.5 cells. Luciferase activity was measured at 4, 24, 48 and 72 hpe and the data were normalized with respect to 4 hpe. N = 3, significant differences from WT denoted by **** (P<0.0001). (PDF) [file ppat.1010812.s001.pdf]

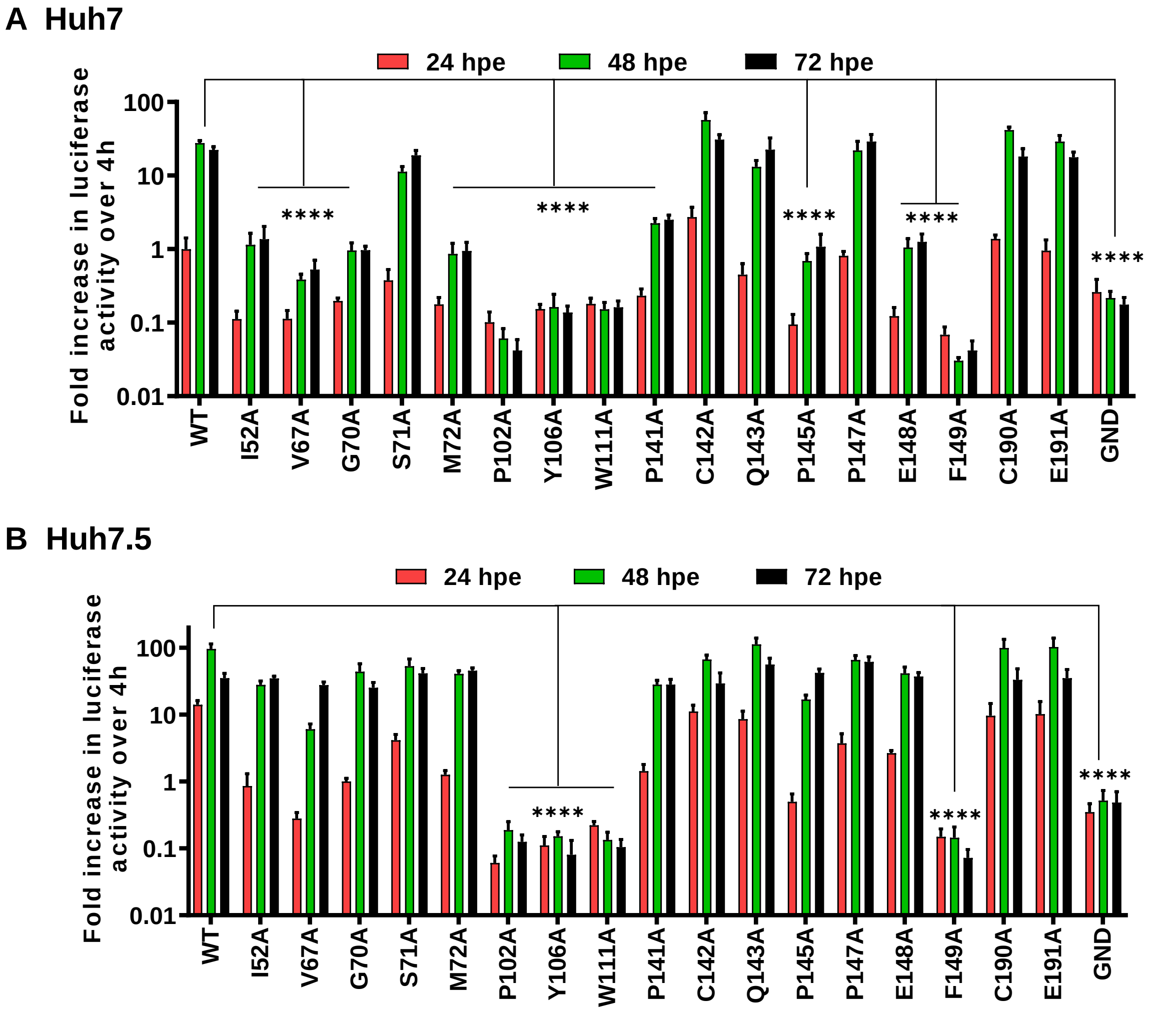

S1 Fig. Genome replication in Huh7 and Huh7.5 cells. *In vitro* transcribed mSGR-luc-JFH-1 RNAs containing the indicated mutations were electroporated into **(A)** Huh7 and **(B)** Huh7.5 cells. Luciferase activity was measured at 4, 24, 48 and 72 hpe and the data were normalized with respect to 4 hpe. N=3, significant differences from WT denoted by **** (P<0.0001).
