# Supplementary material for: NS5A domain I antagonises PKR to facilitate the assembly of infectious hepatitis C virus particles
Source: PLoS Pathog. 2023 Feb 16;19(2):e1010812. doi: 10.1371/journal.ppat.1010812 (PMC9977016; doi:10.1371/journal.ppat.1010812)
Supplement: S2 Fig — Huh7.5 cells were electroporated with mJFH-1 WT and the DI mutant RNAs as indicated, together with an NS5B GND mutant as negative control. Extracellular virus harvested at 72 hpe was titrated in Huh7.5 cells and quantified using the IncuCyte S3. N = 3, significant difference from WT denoted by *** (P<0.001). (PDF) [file ppat.1010812.s002.pdf]

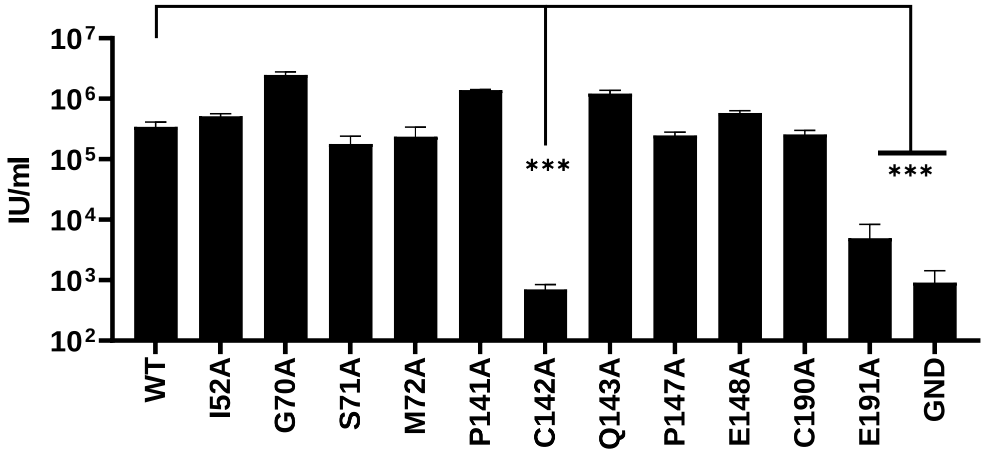

S2 Fig. Virus assembly phenotypes in Huh7.5 cells. Huh7.5 cells were electroporated with mJFH-1 WT and the DI mutant RNAs as indicated, together with an NS5B GND mutant as negative control. Extracellular virus harvested at 72 hpe was titrated in Huh7.5 cells and quantified using the IncuCyte S3. N=3, significant difference from WT denoted by *** ($P<0.001$).
