# Supplementary material for: NS5A domain I antagonises PKR to facilitate the assembly of infectious hepatitis C virus particles
Source: PLoS Pathog. 2023 Feb 16;19(2):e1010812. doi: 10.1371/journal.ppat.1010812 (PMC9977016; doi:10.1371/journal.ppat.1010812)
Supplement: S3 Fig — The three surface exposed residues C142A, C190A and E191A proximal to P145 are displayed in two NS5A DI (genotype 1b) structures 1ZH1 (A) and 3FQM (B). Images on the right are zoomed into the boxed region shown in both space fill and ribbon format. Note that the disulphide bond formed by C142A and C190A was only observed in 1ZH1. (PDF) [file ppat.1010812.s003.pdf]

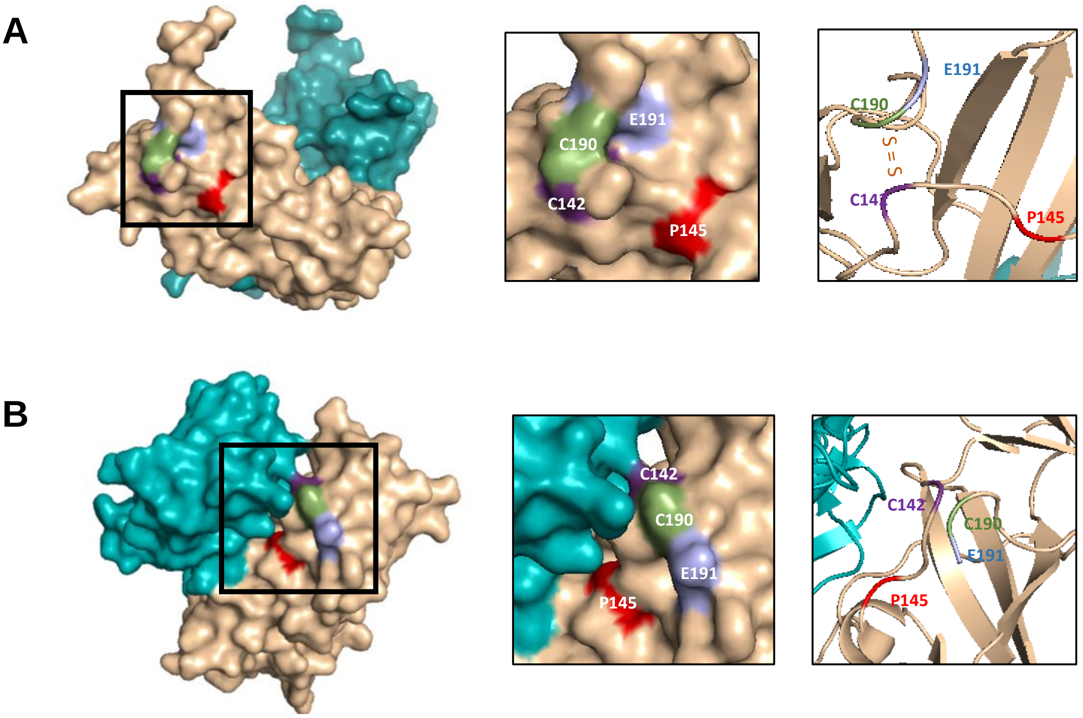

S3 Fig. Location of mutated residues in DI. The three surface exposed residues C142A, C190A and E191A proximal to P145 are displayed in two NS5A DI (genotype 1b) structures 1ZH1 (**A)** and 3FQM (**B).** Images on the right are zoomed into the boxed region shown in both space fill and ribbon format. Note that the disulphide bond formed by C142A and C190A was only observed in 1ZH1.
