# Supplementary material for: NS5A domain I antagonises PKR to facilitate the assembly of infectious hepatitis C virus particles
Source: PLoS Pathog. 2023 Feb 16;19(2):e1010812. doi: 10.1371/journal.ppat.1010812 (PMC9977016; doi:10.1371/journal.ppat.1010812)
Supplement: S4 Fig — Huh7.5 cells were electroporated with mJFH-1 WT and DI mutant C142A, C190A and E191A RNAs, together with an NS5B GND mutant as negative control. TM: tunicamycin positive control. Cells were harvested at 72 hpe and lysed with GLB. eIF2α and phospho-eIF2α was analyzed by western blotting. (PDF) [file ppat.1010812.s004.pdf]

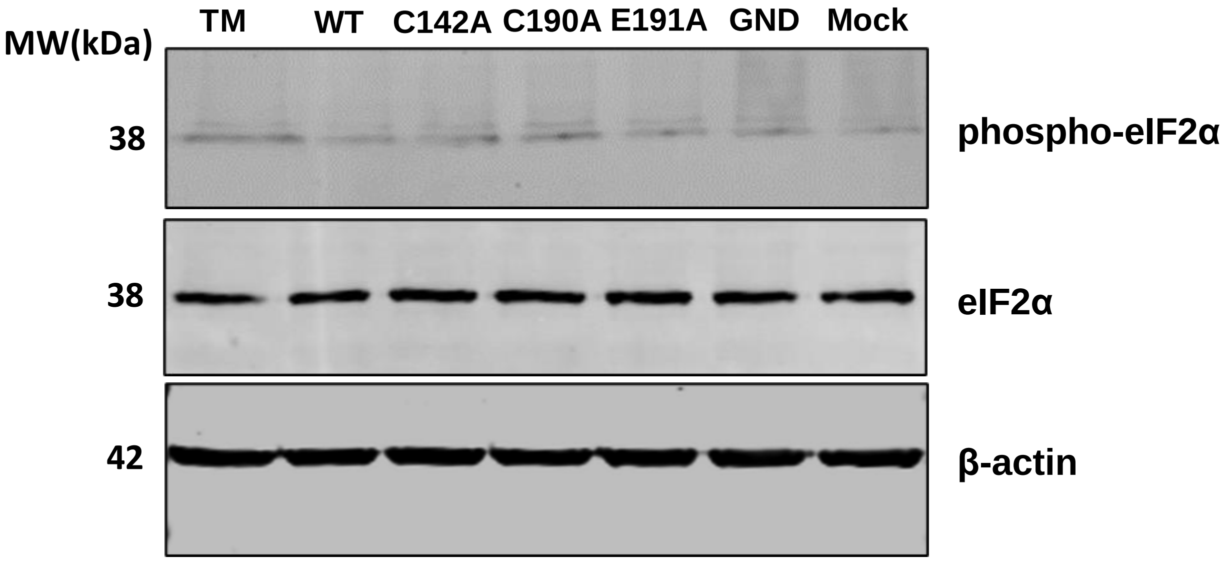

S4 Fig. Expression of eIF2α and phospho-eIF2α. Huh7.5 cells were electroporated with mJFH-1 WT and DI mutant C142A, C190A and E191A RNAs, together with an NS5B GND mutant and mock-electroporated as negative controls. TM: tunicamycin positive control. Cells were harvested at 72 hpe and lysed with GLB. eIF2α and phospho-eIF2α was analyzed by western blotting.
