# Supplementary material for: NS5A domain I antagonises PKR to facilitate the assembly of infectious hepatitis C virus particles
Source: PLoS Pathog. 2023 Feb 16;19(2):e1010812. doi: 10.1371/journal.ppat.1010812 (PMC9977016; doi:10.1371/journal.ppat.1010812)
Supplement: S5 Fig — Huh7.5 cells were electroporated with mJFH-1 WT and DI mutants C142A, C190A and E191A RNAs. At 72 hpe, cells were fixed and stained with mouse anti-P65 (green), sheep anti-NS5A (red) and DAPI. As a positive control to activate the NF-κB pathway uninfected Huh7.5 cells were treated with TNF-α for 24h. Mock: uninfected Huh7.5 cells. (PDF) [file ppat.1010812.s005.pdf]

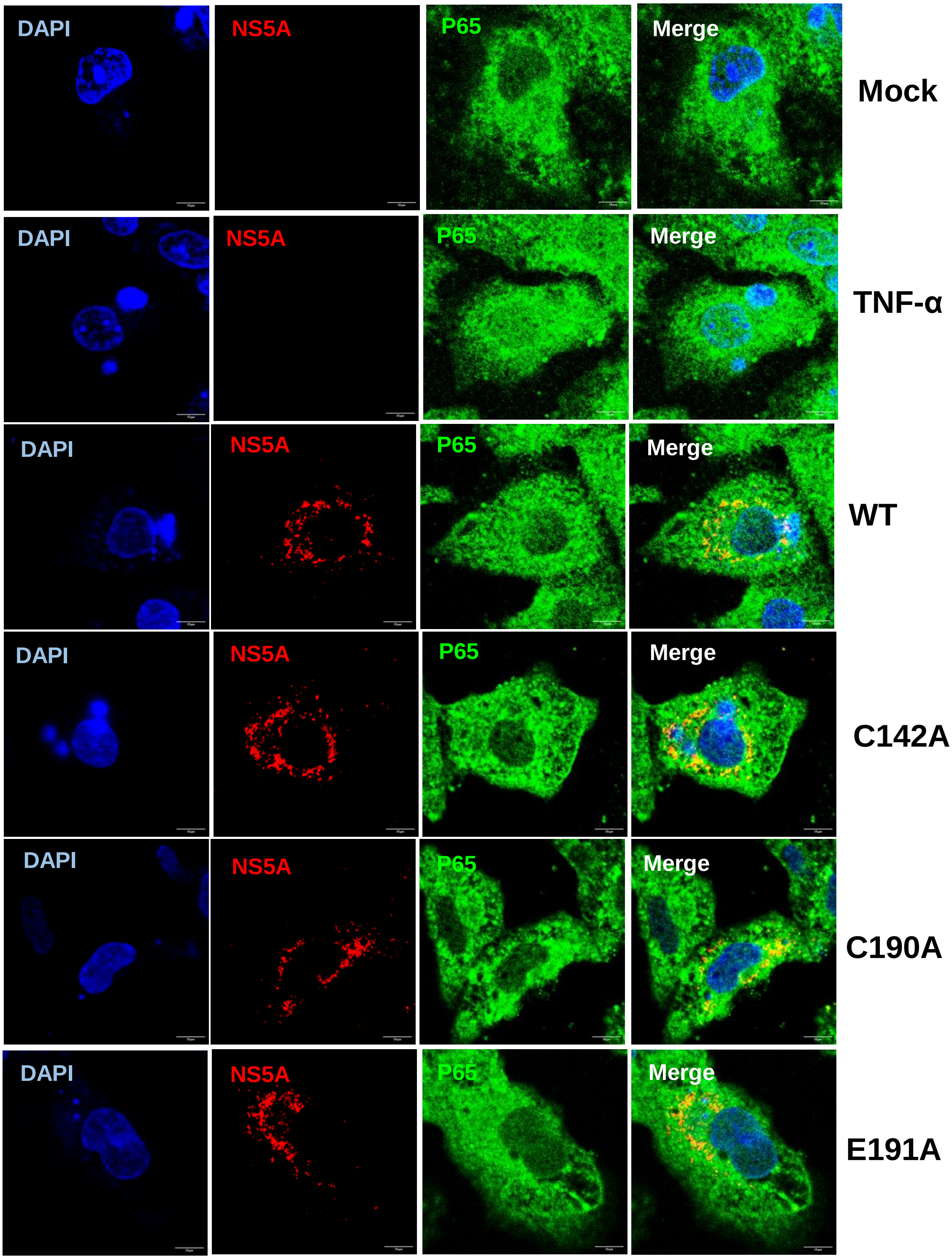

S5 Fig. NF-κB activation in Huh7.5 cells infected with mJFH-1 or DI mutants. Huh7.5 cells were electroporated with mJFH-1 WT and DI mutant C142A, C190A and E191A RNAs. At 72 hpe, cells were fixed and stained with mouse anti-P65 (green), sheep anti-NS5A (red) and DAPI. As a positive control to activate the NF-κB pathway uninfected Huh7.5 cells were treated with TNF-α for 24h. Mock: uninfected Huh7.5 cells.
