# Supplementary material for: NS5A domain I antagonises PKR to facilitate the assembly of infectious hepatitis C virus particles
Source: PLoS Pathog. 2023 Feb 16;19(2):e1010812. doi: 10.1371/journal.ppat.1010812 (PMC9977016; doi:10.1371/journal.ppat.1010812)
Supplement: S6 Fig — (A) Representative image of mock infected cell from Fig 3 stained at 72 hpe with sheep anti-NS5A (white), rabbit anti-Core (green), BODIPY 558/568-C12 (red) and DAPI. Scale bar 5 μm. (B) Representative image of mock infected cell from Fig 7 stained at 72 hpe with sheep anti-NS5A (white), rabbit anti-Core (green), BODIPY 558/568-C12 (red) and DAPI. Scale bar 5 μm. (C) Representative image of mock infected cell from Fig 10 stained at 72 hpe with sheep anti-NS5A (green), mouse anti-dsRNA J2 (white), BODIPY 558/568-C12 (red) and DAPI. (D) Representative image of HCV NS5B GND mutant electroporated cell stained at 4 hpe with sheep anti-NS5A (green), mouse anti-dsRNA J2 (white), BODIPY 558/568-C12 (red) and DAPI. Scale bar 5 μm. (PDF) [file ppat.1010812.s006.pdf]

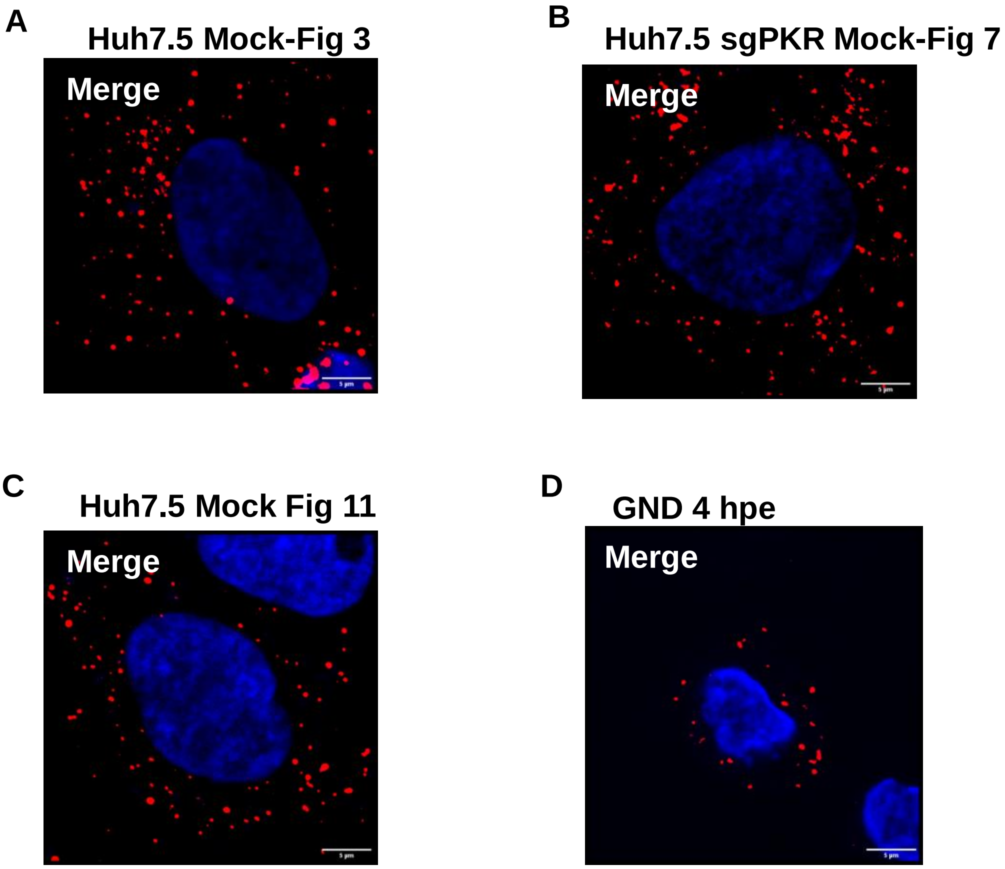

S6 Fig. Control immunofluorescence images. (A) Representative image of mock infected cell from Fig 3 stained at 72 hpe with sheep anti-NS5A (white), rabbit anti-Core (green), BODIPY 558/568-C12 (red) and DAPI. Scale bar 5 µm. **(B)** Representative image of mock infected cell from Fig 7 stained at 72 hpe with sheep anti-NS5A (white), rabbit anti-Core (green), BODIPY 558/568-C12 (red) and DAPI. Scale bar 5 µm. **(C)** Representative image of mock infected cell from Fig 10 stained at 72 hpe with sheep anti-NS5A (green), mouse anti-dsRNA J2 (white), BODIPY 558/568-C12 (red) and DAPI. Scale bar 5 µm. **(D)** Representative image of GND-electroporated cell stained at 4 hpe with sheep anti-NS5A (green), mouse anti-dsRNA J2 (white), BODIPY 558/568-C12 (red) and DAPI. Scale bar 5 µm.
