# Supplementary material for: NS5A domain I antagonises PKR to facilitate the assembly of infectious hepatitis C virus particles
Source: PLoS Pathog. 2023 Feb 16;19(2):e1010812. doi: 10.1371/journal.ppat.1010812 (PMC9977016; doi:10.1371/journal.ppat.1010812)
Supplement: S7 Fig — Huh7.5 cells were electroporated with mJFH-1 WT and DI mutant C142A, C190A and E191A RNAs. The uninfected Huh7.5 cells (Mock) were used as a negative control. The expression of PSMB9 and GAPDH were analysed using qRT-PCR. The relative expression levels of PSMB9 in WT and mutants were compared to mock and shown as Log2 2-ΔΔCt fold change. (PDF) [file ppat.1010812.s007.pdf]

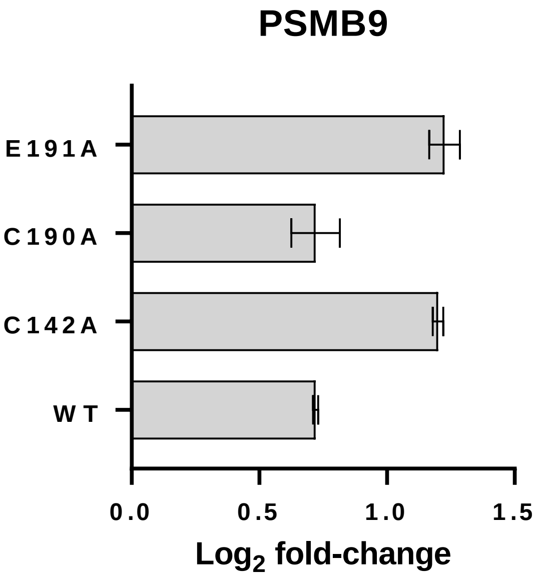

S7 Fig. Expression of an IRF1-responsive gene, PSMB9. Huh7.5 cells were electroporated with mJFH-1 WT and DI mutant C142A, C190A and E191A RNAs. Uninfected Huh7.5 cells were used as a negative control. The expression of PSMB9 and GAPDH were analysed using qRT-PCR. The relative expression levels of PSMB9 in WT and mutant infected cells were compared to uninfected and shown as $\text{Log}_2\ 2^{-\Delta\Delta Ct}$ fold change.
