# Supplementary material for: NS5A domain I antagonises PKR to facilitate the assembly of infectious hepatitis C virus particles
Source: PLoS Pathog. 2023 Feb 16;19(2):e1010812. doi: 10.1371/journal.ppat.1010812 (PMC9977016; doi:10.1371/journal.ppat.1010812)
Supplement: S1 Table — (PDF) [file ppat.1010812.s008.pdf]

Mutant	Quikchange Primer	
I52A	Forward	GGCACTGGCGCGATGACCACGCGCT
	Reverse	GGCCCACACACCCTTGTACCCCTT
G70A	Forward	CGCCTGGCGTCTATGAGGATCACAGGGCC
	Reverse	GACATTGCCAGAGATGTTGGCGC
S71A	Forward	CTGGGCGCGATGAGGATCACAGGGCC
	Reverse	GCGGACATTGCCAGAGATGTTGGCG
M72A	Forward	CTGGGCTCTGCGAGGATCACAGGGCC
	Reverse	GCGGACATTGCCAGAGATGTTGGCG
P102A	Forward	GCGCCGAAAGCGCCCACGAACTACAA
	Reverse	GCACTGGCCCTCCGTGTAGCAAT
Y106A	Forward	CCCCCCACGAACGCGAAGACCGCCA
	Reverse	TTTCGGCGCGCACTGGCCCTC
W111A	Forward	GCCATCGCGAGGGTGGCGGCCTC
	Reverse	GGTCTTGTAGTTCGTGGGGGGTTTC
P141A	Forward	TGAAAATTGCGTGCCAACTACCTTCTCT
	Reverse	ATTGTCAGTGGTCAGTCCTGTTAC
C142A	Forward	CTGAAAATTCCTGCGCAACTACCTTCTCCAG
	Reverse	ATTGTCAGTGGTCAGTCCTGTTAC
Q143A	Forward	CCTTGCGCGCTACCTTCTCCAGAGTTT
	Reverse	AATTTTCAGATTGTCAGTGGTCAGTC
P147A	Forward	CCTTCTGCGGAGTTTTTCTCCTGGG
	Reverse	TAGTTGGCAAGGAATTTTCAGATTGTC
E148A	Forward	CCTTCTCCAGCGTTTTTCTCCTGGGTGG
	Reverse	TAGTTGGCAAGGAATTTTCAGATTGTC
F149A	Forward	CCAGAGGCGTTCTCCTGGGTGGACGG
	Reverse	AGAAGGTAGTTGGCAAGGAATTTTCAG
C190A	Forward	CAGCTTCCCGCGGAACCTGAGCCCGA
	Reverse	GGACCCGACAGCATAGGAATTAAG
E191A	Forward	CAGCTTCCCTGTGCGCCTGAGCCCGA
	Reverse	GGACCCGACAGCATAGGAATTAAG
**Protein expression**	**Oligonucleotide Sequences**	
His-Sumo-NS5A DI 35-215 (WT C142A C190A and E191A)	Forward	CTGCCCGGATCCCCCTTCATCTCTTGTCAAAAGGG
	Reverse	CGTGCCCTCGAGTTACGCCGCAGTCTCCGCCGTG
pCDNA3.1-PKR	Forward	CGCGGATCCATGGCTGGTGATCTTTCAGCA
	Reverse	CCGCTCGAGCTAACATGTGTGTCGTTCATTTT
**qRT-PCR**	**Oligonucleotide Sequences**	
HCV	Forward	TCTGCGGAACCGGTGAGTA-
	Reverse	TCAGGCAGTACCACAAGGC
PSMB9	Forward	GTGGATGCAGCATATAAGCC
	Reverse	AGTGACCAGGTAGATGACAC

S1 Table: oligonucleotide sequence of primers used in this study
